# Registered report protocol: Developing an artifact index for capacitive electrocardiography signals acquired with an armchair

**DOI:** 10.1371/journal.pone.0254780

**Published:** 2021-07-28

**Authors:** Joana M. Warnecke, Ju Wang, Tolga Cakir, Nicolai Spicher, Nagarajan Ganapathy, Thomas M. Deserno

**Affiliations:** Peter L. Reichertz Institute for Medical Informatics of TU Braunschweig and Hannover Medical School, Braunschweig, Germany; Kuwait College of Science and Technology, KUWAIT

## Abstract

Continuous monitoring of an electrocardiogram (ECG) in private diagnostic spaces such as vehicles or apartments allows early detection of cardiovascular diseases. We will use an armchair with integrated capacitive electrodes to record the capacitive electrocardiogram (cECG) during everyday activities. However, movements and other artifacts affect the signal quality. Therefore, an artifact index is needed to detect artifacts and classify the cECG. The unavailability of cECG data and reliable ground truth information requires new recordings to develop an artifact index. This study is designed to test the hypothesis: an artifact index can be devised, which intends to estimate the signal quality of segments and classify signals. In a single-arm study with 44 subjects, we will record two activities of 11-minute duration: reading and watching television. During recording, we will capture cECG, ECG, and oxygen saturation (SpO_2_) with time synchronization as well as keypoint-based movement indicators obtained from a video camera. SpO_2_ provides additional information on the subject’s health status. The keypoint-based movements indicate artifacts in the cECG. We will combine all ground truth data to evaluate the index. In the future, we aim at using the artifact index to exclude cECG segments with artifacts from further analysis. This will improve cECG technology for the measurement of cardiovascular parameters.

## Introduction

According to the World Health Organization (WHO), cardiovascular diseases (CVD) cause 17 million deaths annually [1]. Continuously monitoring of cardiovascular parameters in private spaces, such as vehicles and apartments, bears the potential of earlier detection of CVD symptoms and diseases [2], which could improve the therapeutic outcome and decrease mortality [3, 4]. The activity of the heart muscle cells generates an electric excitation that yields an electric potential on the body surface, which can be measured conventionally as an electrocardiogram (ECG) or indirectly as capacitive coupled ECG (cECG). As a major advantage, a cECG records through clothes without the need for body surface contact. Authors reported various applications in vehicles [5–7] and apartments, for example in a bed [8–10] or chair [11–13]. In western countries, people watch television for about 3.5 hours per day [14, 15]. Hence, the living room enables unobtrusive and continuous monitoring of the cardiovascular system. We embedded cECG electrodes in an armchair and integrated the recording device in the smart home bus system [16].

However, the signal-to-noise ratio (SNR) of cECG is affected by several factors, such as movement artifacts [13, 17], environmental electromagnetic interferences [17], and variation of the coupling impedance [18]. To increase the reliability and accuracy of cardiac parameters, signal quality assessment (SQA) plays a pivotal role: It allows to determine the periods, in which the SNR is adequate for accurate measurement of cardiovascular parameters. Typically, such parameters are derived from fiducial points and their course over time [19]. The most prominent fiducial point is the R wave, which is part of the QRS complex that represents the contraction of the heart.

The distance between two consecutive R waves is denoted RR-interval (RRI) and its variation over time is termed heart rate variability (HRV), which indicates cardiac abnormalities and other CVDs [20]. The heart rate (HR) itself denotes the number of heartbeats per minute [20].

In the literature, different approaches have been proposed for SQA assessment of clinical ECG [19]. Satija et al. identified five categories of SQA-based methods: (i) heuristic rules based on fiducial features, (ii) machine learning based on fiducial features, (iii) heuristic rules based on non-fiducial features, (iv) machine learning based on non-fiducial features, and (v) filtering techniques [19]. However, the measurement principle of capacitive electrodes differs fundamentally from adhesive contact electrodes [21]. The cECG signal has a different morphology and signal features [21], and SQA approaches for ECG cannot be directly applied to cECG. So far, SQA approaches of cECG signals have only been proposed rarely [18, 22].

For example, Hou et al. measured six subjects with four capacitive electrodes attached to an office chair [13]. Based on phase-space reconstruction, they developed an SQA approach to aggregate multiple signals. The signal quality index was evaluated with data from the MIT-BIH arrhythmia database, which contains long-term ECG recorded with a Holter monitor [12]. It was shown that the index could indicate the quality change of these ECG signals as well, despite the difference to cECG. Multiple paper investigated the reduction of artifacts during measurements. In 2011, Wartzek et al. developed cECG electrodes to reduce the triboelectric effect [17] and tested the robustness of cECG electrodes for unobtrusive monitoring with 59 car drivers [7]. The measurements in city traffic had a lower SNR than those on the highway. During city and highway traffic, 6%—61% and 65%—86% of the driving time contained at least a segment of four consecutive R waves without artifacts, respectively. The authors also developed five detection algorithms for artifacts and tested sensitivity and specificity. The approach based on the Hotelling’s T-squared values achieved the best results. Additionally, the approach compared the minimum and maximum amplitudes to detect false R waves [7]. These values present a confidence measure for ECG segmentation, which depends on the SNR of a data set. Fukuyama et al. investigated multi- and single-layered electrodes to reduce movement artifacts during cECG measurements [22]. They showed that multi-layered fabric electrodes reduce movement artifacts. Wang et al. used flexible capacitive electrodes attached to a chest belt and tested the impact of different clothing with a SQA approach based on template-matching [23].

According to Hou et al., Fukuyama et al., and Wartzek et al., the SNR of cECG is affected by: (i) shape of the body, (ii) movement, (iii) electromagnetic disturbance, (iv) thickness of the cloth and material, (v) triboelectric effect, and (vi) electromagnetic interferences [13, 17, 22]. Unfortunately, the cECG data from these studies are unavailable. However, the UnoViS database contains cECGs from six volunteers recorded during driving [24] (unpublished recordings of [7]). A driving car is incomparable to a stationary chair in an apartment.

The lack of publicly available data makes the development of SQA methods cumbersome. Within this study, we will record cECG data from an armchair with integrated electrodes. Using this data, we will (i) analyze whether the given factors influencing SNR [13, 17, 22] and ii) develop an artifact index customized for cECG.

We will propose a SQA method and evaluate it on ECG and peripheral capillary oxygen recorded saturation (SpO_2_) as ground truth in parallel. To identify movement artifacts, we record the positions and poses of the subjects with a video-based keypoint extraction method [25]. The output of the SQA method will serve as an artifact index, which classifies the signal in signal quality classes.

## Materials and methods

### Measuring system

All data will be recorded in a furnished research apartment at TU Braunschweig. It provides an open living and kitchen area, a bathroom, and a bedroom. The cECG armchair is located in the open living area in front of the television. The temperature is constantly regulated to 21 degrees Celsius during the recording. The average humidity is included in the metadata. In addition to the cECG signal, ground truth recordings are composed of ECG, SpO_2_, and keypoint-based movements.

#### cECG armchair

**Fig 1:**
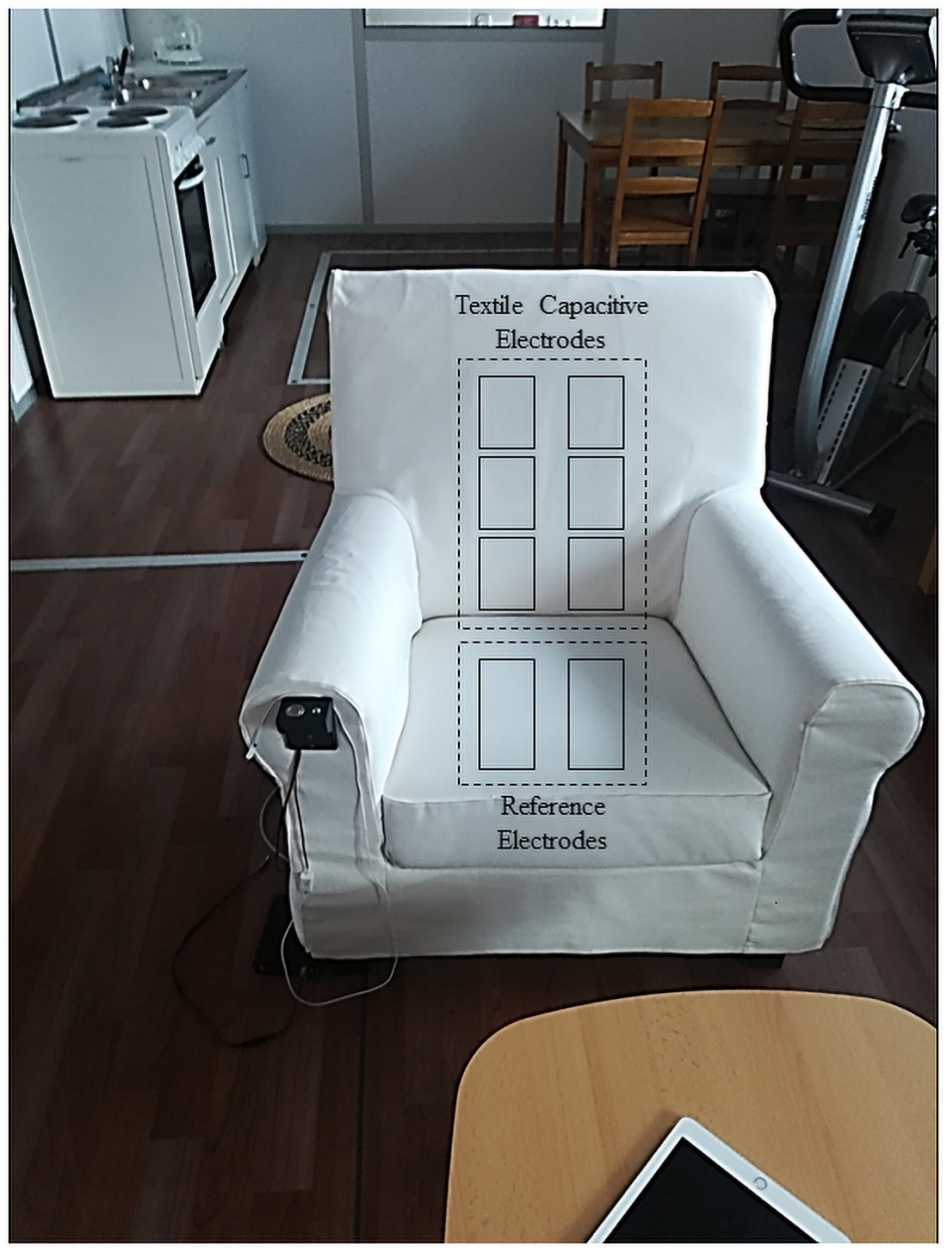
cECG armchair with capacitive electrodes.

In this study, we will use a cECG system (prototype, Capical GmbH, Braunschweig, Germany), which records signals with a 560 Hz sampling rate and 16-bit resolution with eight textile capacitive electrodes embedded under the chair cover. Six capacitive electrodes are installed in the backrest and two reference electrodes are located in the seat cushion (Fig 1).

**Fig 2:**
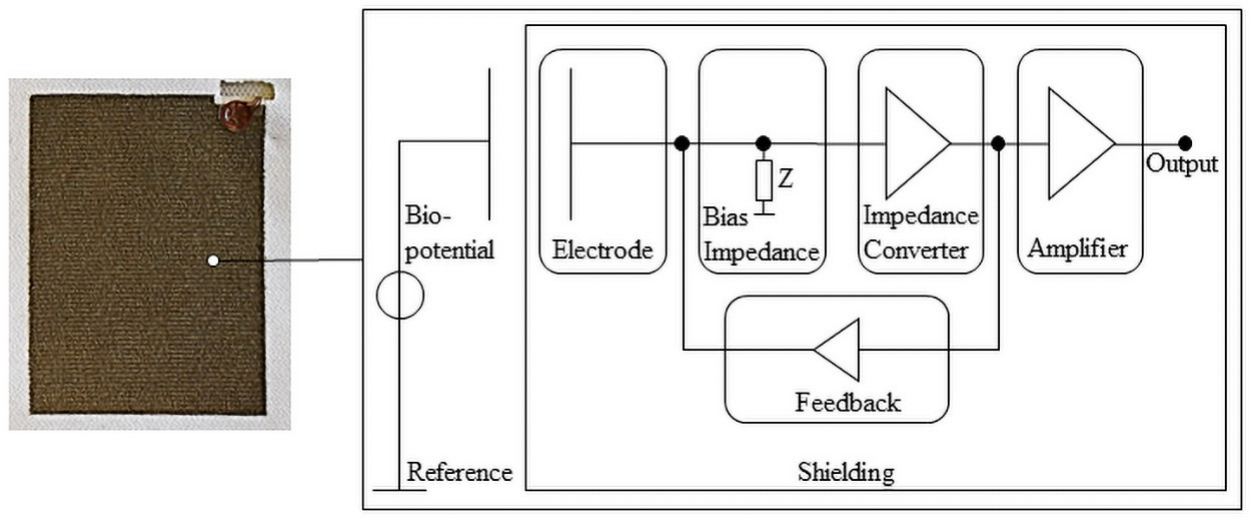
Schematic diagram of textile capacitive electrodes [21].

The capacitive electrodes are textile and active electrodes based on silver yarn and polymer-based fiber with a woven structure. The metallic element silver and woven structure improves the surface conductivity [26]. The basic structure of the textile capacitive electrode consists of the electrode, impedance (Z) converter, amplifier, and shielding (Fig 2). On the left-hand side is shown the textile capacitive electrode and on the right-hand side the block diagram.

The flexibility of the textile electrodes used in this study improves the contact during movements. The comfort of the test person for long-term recordings is also higher because textile electrodes are more flexible and stretchable.

#### ECG reference data

**Fig 3:**
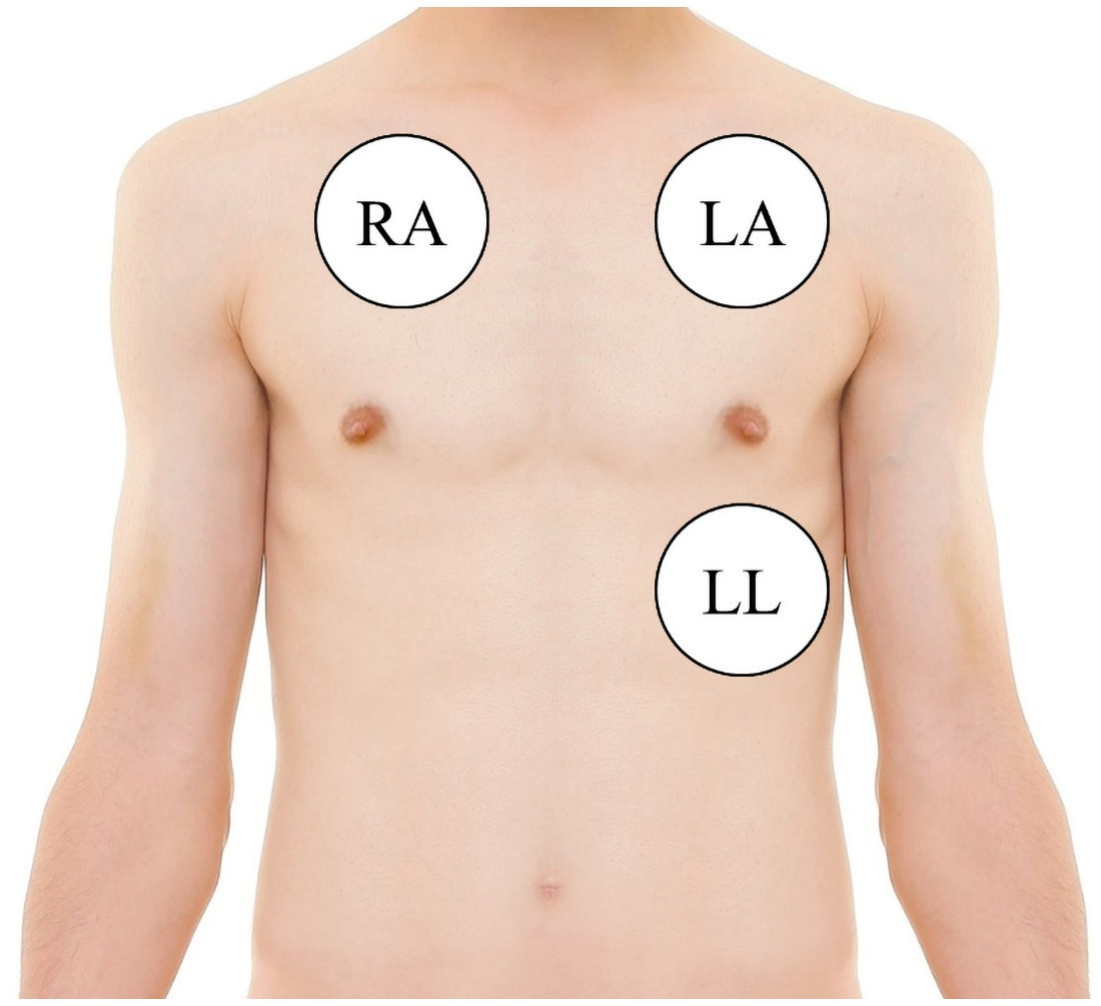
Position of adhesive electrodes for ECG recording.

An ECG sensor (BiosignalPlux Explorer, Plux Wireless Biosignals, Lisboa, Portugal) will record the ground truth with a 500 Hz sampling rate and 16-bit resolution. The adhesive electrodes will be attached to three positions of the upper body (Fig 3):

- RA (right arm/positive): central, just below the right collarbone
- LA (left arm/ground): central, just below the left clavicle
- LL (left leg/negative): left, middle clavicular line in the fifth intercostal space

#### Peripheral capillary oxygen saturation

A SpO_2_ sensor (BiosignalPlux Explorer, Plux Wireless Biosignals, Lisboa, Portugal) will record the peripheral capillary oxygen saturation also with a 500 Hz sampling rate and 16-bit resolution. Referring to the manufacturer’s specifications, the accuracy is ± 2% from a clinical device. According to Basaranoglu et al., the sensor will be applied to the right-handed middle finger during the measurement [27].

#### Keypoint-based movements

We will extract keypoints from a video, which describes the pose of the subject sitting in the armchair using an open-source implementation of the PifPaf algorithm [25]. It is a bottom-up human pose detector, which determines the spatial coordinates based on body landmarks (for example arms, legs, and back) from a person. Only the keypoints will be stored and subject identification is not possible. The PifPaf algorithm delivers 2D coordinates of keypoints of the test person and does not automatically mark the movements. However, by analyzing the estimated pose in sequential frames we can derive movements. The camera module (camera v2.1, Raspberry Pi Foundation, Cambridge, England) will record the video with 10 frames per second (FPS).

#### Data synchronization

To timely synchronize cECG, SpO_2_, and ECG, an optical synchronization cable (BiosignalPlux Explorer, Plux Wireless Biosignals, Lisboa, Portugal) will be used. The adjustable sampling rate will be set to 500 Hz, with a 16-bit resolution. The keypoints will be synchronized by the video synchronization add-on from Plux.

## Study design

### Hypothesis

The study is designed to test the hypothesis: an artifact index can be devised, which intends to estimate the signal quality of 10-second segments and to classify the signal in one of five signal quality classes: 1. excellent >95%, 2. good 95 − 85%, 3. adequate 85 − 75%, 4. poor 75 − 60%, and 5. unacceptable <60% based on [28]. For example, if 10 seconds of a 60-second cECG contain artifacts, 50/60 = 83.3333% of the signal are without artifacts and the signal belongs to signal quality class 2. Artifacts corrupt the cECG signal and can cause wrong diagnostics, for example, an artifact caused by movements.

Primary question (PQ):

- PQ1: Which signal quality classes of the artifact index are appropriate to calculate the heart rate?

Secondary question (SQ):

- SQ1: Which factors affect the data quality of the cECG signal?
- SQ2: Does the cECG chair cause significant signal-to-noise ratios that can be identified as noise?

Side observation (SO):

- SO1: Which cECG leads show the most reliable measured values?
- SO2: Is it possible to distinguish different artifacts?

### Type of study

This is a single-arm observational pilot study.

### Inclusion criteria

The inclusion criteria are:

1. older than 18 years, and
2. no previous illnesses with permanent impairments.

#### Exclusion criteria

The exclusion criteria are:

1. pacemaker,
2. heart diseases such as atrial fibrillation (AF),
3. infectious diseases such as methicillin-resistant staphylococcus aureus (MRSA),
4. open wounds, and
5. prominent hair growth on the torso.

### Identification of the inclusion and exclusion criteria

We will assess if a subject matches the inclusion and exclusion criteria. The assessment will be conducted by instructed employees, who interview the subjects.

### Measurement protocol

Within the study, two consecutive stages of at least eleven minutes will be conducted while the subject is sitting comfortably in the chair and relaxes. First, the subjects will read a book or magazine and then they will watch television. We consider these stages as everyday activities.

### Preparation of the subject

The subjects themselves will attach three adhesive electrodes to their body in the bathroom, according to a pictured description (Fig 3). To obtain comparative data, all subjects will wear a T-shirt (100% cotton). Before starting the measurement, we will verify that all signals are recorded appropriately. The integrated electrodes in the cushion are reference electrodes for grounding. However, the material of the pants can have an impact on the signal quality. Therefore, we will list the type of trousers in the metadata of each recording.

### Effect of clothing material

To show the effect of different clothing material on the lower body, we will conduct an additional measurement with five subjects. The measurement system and study protocol are identical. Wang et al. tested the different materials: a. cotton, b. flax, c. jeans, and d. polyester [23]. For comparison, we will test the same clothing material.

### Demographic data

All subjects will provide information about their age, height, weight, and gender.

### Study time line

The study will end after 16 weeks. We will share the study data and results with all subjects and the scientific community.

### Data management, security, and privacy

Demographic and measured data will be stored on a server of the Peter L. Reichertz Institute (PLRI) at TU Braunschweig using OpenClinica version 2.1 (OpenClinica, LLC, Waltham, MA, USA). All data transfer and communication with the servers is encrypted via HTTPS (TLS 1.2, 256-bit key) or SSH.

Physical access to the servers is only permitted to selected personnel and requires a transponder. Individual data will be linked to a subject’s pseudonym. This identifier (ID) is known and recorded only by the subjects herself or himself. The ID is not displayed to the study personnel. Providing the ID and the corresponding recording date, a subject can request the deletion of the data without giving reasons.

### Data sharing

Neither cECG, ECG, SpO_2_ nor the keypoint-based two-dimensional coordinates store any information allowing to deduce the subject’s identity. The anonymous data will be accessible over the data storage system of TU Braunschweig. The data we want to share is completely anonymous. Therefore, the *General Data Protection Regulation* (GDPR) does not apply and the German legal regulations allow the publication of anonymous data. We will share the following data with the comma-separated values (CSV) file format:

- demographic data (age, height, weight, and gender),
- metadata (humidity and type of trousers),
- cECG data,
- ECG reference data,
- peripheral capillary oxygen saturation, and
- 2D coordinates of keypoints.

However, the subjects can request the deletion of their data. For this reason, everyone has to request the data from the corresponding author per e-mail. After approval, this person can download the data from the data storage system of TU Braunschweig. If a subject requests the deletion of the data with the ID and the corresponding recording date, we will delete the data from the data storage system of TU Braunschweig. Additionally, we will contact all persons, who requested the data and we will request the deletion of the data set with the corresponding ID. Therefore, we chose the license type *All Rights Reserved* instead of *CC BY*.

### Subjects

#### Safety of subjects

During the study, all measurements are painless, non-invasive, and carry no substantial risk.

#### Sample size

The presented study is a single-arm observational pilot study. Accordingly, the sample size yields [29]:

$$\begin{array}{c} n=\frac{4\cdot z^{2}\cdot p\cdot \left(1-p\right)}{\omega^{2}} \end{array}$$ (1)

Where *n* denotes the number of subjects. Assuming a standard normal distribution, the z-score is determined for a 95% confidence interval (*z* = 1.645). The p-value stands for the probability that the derived HR of the cECG is equal to the reference ECG. For medical applications, we require the correct HR with 80% (*p* = 0.8). However, this probability only applies to cECG signals with an appropriate signal quality class. The width of the confidence interval is denoted by *ω*. Due to the pilot character of the study, we choose *ω* = 0.2 [29]. Therefore, (1) yields:

$$\begin{array}{c} n=\frac{4\cdot 1.645^{2}\cdot 0.8\cdot \left(1-0.8\right)}{0.2^{2}}=43.2964 \end{array}$$ (2)

and the number of required subjects is 44.

#### Recruitment of subjects

Subjects will be recruited from academia (students, scientists, teachers, and university employees) at both campuses of PLRI: TU Braunschweig and Hannover Medical School, Germany. The volunteers will not be rewarded financially for their engagement. They will be informed about the study goals by documents, verbal communication, and the PLRI web pages. If the subject does not meet any exclusion criteria, patient information, and consent will be provided. The principal investigator and trained employees are responsible for interviewing the subjects, confirming their consent, and answering possible questions. Study participation does not affect the subjects and does not result in any discrimination or preference.

### Adverse events

All adverse events (for example, wet cleaning of the chair due to stains) will be documented including the time of occurrence, type, duration, intensity, and frequency. All other technical or organizational confounding factors that may influence the study results are documented.

### Technical disorders

If the data recording is interrupted by software or hardware failures, the recording will be repeated.

### Organizational disorders

Organizational disturbances include all errors and problems that are not directly related to the technology used in the study. Organizational disturbance variables include a lack of documentation and time delays. Due to possible organizational disturbances, errors can occur in the study documentation (for example loss, confusion of subjects, and data, etc.), which can reduce the significance of the overall study.

## Regulations

### Data security

The data security concept has been approved by the data security officer of TU Braunschweig (reference number: PscECG1).

### Ethic

The study protocol has been approved by the MHH ethics committee (reference number: 9287 BO S2020) [30]. The study will be performed in conformance with the Helsinki declaration [31].

### Conflicts of interest

For PLRI, there is a fundamental interest in developing a suitable artifact index to determine cECG quality. However, this will not be influenced by the consideration of the diligence of the scientific execution. The objectivity of the study can be guaranteed by a critical consideration and the extensive and division of implementation, analysis, supervision, and monitoring by study staff, as well as the adherence to the premise of the dual control principle, as well as universal study transparency.

## Discussion

### Expected outcome

The cECG, ECG, SpO_2_, and keypoint-based movements from 44 subjects and their demographic data will be part of the outcome of this study. They will be used to develop a cECG artifact index. The artifact index will allow a cECG classification in five signal quality categories based on 10-second segments. If a segment contains artifacts, not all heartbeats are detectable. Such periods will be blended out from the further signal analysis.

The recording of ECG reference and SpO_2_ data will provide a reliable ground truth. Besides, it will enable the monitoring of HRV as well as pulse rate [32]. It will also provide more comprehensive information on the health status of a subject, for example, a low pulse rate and a high HRV indicate a good state of health [32].

Also, the keypoint-based movement assessment will explain detected artifacts. In the future, we aim at using only those segments of a cECG that have an appropriate artifact index class. This will improve cECG technology to detect cardiovascular diseases.

During the design phase, we also considered accelerometer. However, to identify the movements with accelerometers, we would have to attach multiple accelerometers to the body parts that are intended to be monitored. For example, the arms, legs, and hands, as well as the head. In our pre-tests, the camera in combination with human pose estimation delivered accurate results for movement identification. To simplify the data collection, we decided to use a camera only to measure body movements. We selected a camera-based method because an image contains more information for human activity recognition and movement detection than an accelerometer [33].

### Technological developments

In the following, we discuss the properties of the cECG armchair in light of recent technological developments proposed in other works. In 2012, Beak et al. installed capacitive electrocardiogram electrodes, ballistocardiogram, and photoplethysmogram into a diagnostic chair [34]. Two electrodes are placed on the back and a driven capacitive ground circuit in the cushion under the right leg. The electrodes have an aluminum shielding case due to the surface conductivity. Ahn et al. integrated dry-contact capacitive coupling electrodes, and a ballistocardiogram into an office chair [35]. Two electrodes of the capacitive electrocardiogram are placed in the back and one in the center of the cushion. Also, the three electrodes have an aluminum shielding case. Hou et al. placed four electrodes, which are also surrounded by a metal shielding case in the back and the reference electrode is larger–it covers half of the cushion [13]. These are rigid electrodes with a massive metal surface, which are more sensitive for movement artifacts, and new materials were developed. To improve skin contact during movements, we choose textile and flexible electrodes. The number, as well as the size of the capacitive electrodes from the used cECG chair is higher. The higher number of electrodes increases the number of leads. To decrease the skin contact impedance, the cECG chair has a higher electrode area than previous studies [13, 35] and a woven structure.

In 2016, Sun et al. reviewed capacitive measurements to reflect technological developments in improving the signal quality [36]. They discussed the aspects of material selection, dimension design, and electrode array as these parameters have a considerable impact on signal quality [36]. The material selection determines the conductivity of the electrode. In their review, they highlighted a paper from Hoffmann et al. [37], who compared 16 materials and electrodes made of a conductive polymer layer achieving the best results. The reason lies in the polymer structure enabling more flexibility and improving contact during movements. The capacitive electrodes of our cECG armchair also have a polymer-based fiber structure with silver yarns. The dimension design includes the different designs of the electrode surface. Sun et al. recommended a design, which enables a high comfort [36]. The cECG armchair used in this study has a woven structure, which enables high comfort as well. Regarding the electrode array, Sun et al. underlined the importance of redundant information realized by a higher number of electrodes and leads [36]. The cECG armchair has six capacitive electrodes in the backrest and two reference electrodes, which delivers redundant information.

Pani et al. summarized materials and fabrication technologies used in textile electrodes [38]. They defined key characteristics of textile ECG electrodes as conductivity, skin contact impedance, morphological features, comfort, and washability [38]. They pointed out that metallic elements increase the surface conductivity and underlined the importance of conductive polymers. Besides, Pani et al. show that silver has a higher conductivity than copper, bronze, and steel [38]. This concept is also considered when developing the textile electrodes for our cECG armchair. The skin contact impedance is affected by skin properties and technical features [38]. For instance, a smaller electrode area increases the skin contact impedance. In addition, the structure of the material is relevant. Pani et al. underlined the high importance of the visibility of morphological features in the cECG signal for identifying clinically relevant fiducial points. We cannot answer the visibility of morphological points at this point but will apply a state-of-the-art ECG delineation algorithm as a subset after completion of the study [39]. Flexibility and stretch enable comfort for long-term recordings and washability guarantees feasibility in daily living conditions. The cECG armchair used in this study has an additional textile layer and it is possible to clean the chair with water.

In another work, Peng et al. compared three active electrode materials: copper foil tape, conductive textile, and flexible printed circuit (FPC) [40]. They reported that using all tested materials the waveform was visible [40]. They used two electrodes but suggested using more signal channels and adding more electrodes as this makes the electrodes more resistant against movements. In our recording system, we will use six electrodes.

In 2021, Wang et al. published a paper about capacitive electrodes with negative impedance to overcome the mismatch of impedance between skin and electrode [41]. In their study, they showed the reliability of negative impedance for non-contact measurements. The SNR was improved from 23.9 dB to 30.2 dB and the maximum cloth-thickness was 1.8 mm [41]. This is a promising approach for future electrode design that could be addressed by modifying the cECG chair for future experiments.

### Limitations

#### Activities

In this study, we only consider two activities, namely reading and watching television in a comfortable and relaxing position. Thereby, both arms can be expected to mostly rest on the armrest. This differs from other daily-life activities, such as talking, eating, or playing video games.

#### Variability of factors

Besides, the subjects will only wear a t-shirt. In a smaller group, we will test different clothing for the lower body. Besides, the chair will be cleaned after each recording, for example, fibers from clothing will be removed. Wet cleaning, i.e., with water and a microfiber cloth, is necessary when stains get on the chair. Nevertheless, the next measurement will be continued only when the chair is dry. Due to the better conductivity of a wet surface, the results will be improved [42]. The skin contact impedance is an important factor [42]. Skin properties of individuals have a large variability and change over time based on the temperature, skin humidity, activity, and sweating [42, 43]. Due to the lower resistance has sweating a positive impact on the measurement [42]. Climate control regulates the temperature to 21 degrees Celsius. The humidity is recorded in the metadata.
